# Knowledge and Use of Novel Psychoactive Substances in an Italian Sample with Substance Use Disorders

**DOI:** 10.3390/ijerph19020915

**Published:** 2022-01-14

**Authors:** Deborah Dal Farra, Alice Valdesalici, Giancarlo Zecchinato, Alfio De Sandre, Diego Saccon, Pierluigi Simonato, Ornella Corazza, Giovanni Martinotti, Andrew L. Smith, Marco Solmi

**Affiliations:** 1 Department of General Psychology, University of Padua, 35122 Padova, Italy; deborahdalfarra@gmail.com (D.D.F.); valdesalici.alice@gmail.com (A.V.); 2 Addiction Department, AULSS 6 Euganea, 35131 Padova, Italy; giancarlo.zecchinato@aulss6.veneto.it; 3 Addiction Department, AULSS 1 Dolomiti, 32021 Agordo, Italy; alfio.desandre@aulss1.veneto.it; 4 Addiction Department, AULSS 4 Veneto Orientale, 30027 San Donà di Piave, Italy; diego.saccon@aulss4.veneto.it; 5 Parco dei Tigli, 35037 Teolo, Italy; pierluigi.simonato@gmail.com; 6 School of Life and Medical Sciences, University of Hertfordshire, Hatfield AL10 9AB, UK; o.corazza@herts.ac.uk (O.C.); giovanni.martinotti@gmail.com (G.M.); 7 Department of Neuroscience, Imaging, Clinical Sciences, University “G.d’Annunzio”, 66100 Chieti-Pescara, Italy; 8 Department of Psychiatry, University of Ottawa, Ottawa, ON K1N 6N5, Canada; andrewlsmith@toh.ca; 9 Department of Mental Health, The Ottawa Hospital, Ottawa, ON K1H 8L6, Canada; 10 Clinical Epidemiology Program, Ottawa Hospital Research Institute (OHRI), University of Ottawa, Ottawa, ON K1N 6N5, Canada

**Keywords:** novel psychoactive substances, substance use disorder, addiction services, prevalence, risk factors

## Abstract

This study aims to determine prevalence and frequency of use of novel psychoactive substances (NPS) and to identify the factors associated with NPS use in an Italian sample of patients diagnosed with substance use disorder (SUD). Prevalence and correlates of NPS knowledge and use were assessed in 185 patients with SUD in three addiction services (Padova, Belluno, Feltre) in the Veneto region with an ad-hoc designed survey. Two thirds of the samples reported knowing NPS and one third reported using them. NPS were considered by them less dangerous than “regular” substances of abuse (t = 6.06 mean 0.78, *p* < 0.001). Factors associated with NPS use were youth (OR = 4.81; *p* < 0.001), studentship (OR = 4.99; *p* = 0.004), subsequent mental disorders diagnosis (OR = 2.49; *p* = 0.027), suicide attempt history (OR = 11.67; *p* < 0.001), home detention (OR = 2.30; *p* = 0.040), residential care (OR = 5.66; *p* = 0.002), and polysubstance abuse (t = 8.99 mean 2.65 *p* < 0.001). NPS use in patients with SUD is highly prevalent, particularly in the youngest age group, and associated with psychiatric comorbidity and worse prognosis. It is crucial to systematically assess NPS use and inform addiction service users with SUD of the toxic and potentially lethal side effects. Mental healthcare professionals working in addiction services should receive education and training. Cohort and longitudinal studies are needed.

## 1. Introduction

Drug abuse is a public health issue that policy-makers and governmental authorities have wrestled with internationally for decades. Increasingly, new psychoactive substances (NPS) are becoming designed and distributed on the illicit market, complicating the already challenging situation. According to relevant legislation, a NPS is any new substance, either psychotropic or neurotic, that is not controlled by the international drug conventions but could have similar aversive and threatening effects of traditional drugs [1,2]. A NPS could be a newly synthetized substance that mimics the effects of more established illegal drugs or a substance already developed and used in other contexts, such as medication that has begun to be consumed for recreational purposes [3]. These new drugs are introduced on the market as “legal” alternatives to more commonly used drugs, such as heroin, cocaine, marijuana, and LSD, thanks to the fact that, as implicit in their definition, they are often not classified under the tables of illegal substances by the authorities [1]. In addition, NPS are mostly undetectable through traditional drug tests and easily distributed over the internet, especially via the dark web [4,5,6]. NPS are also known as designer or synthetic drugs, research chemicals, smart drugs, and, most commonly, legal highs [3,6,7]. The list of novel substances on the drug market is growing exponentially, with approximately 1004 NPS being reported to the United Nations Office on Drugs and Crime (UNODC) and 830 NPS being reported to the European Monitoring Centre for Drugs and Drug Addiction (EMCDDA) by the end of 2020 [8,9]. NPS can be classified into four major groups, namely synthetic stimulants, depressants, cannabinoids, and hallucinogens, together with substances of natural origin [4,10]. According to self-reported data, it is estimated that the one-year prevalence of NPS use among individuals aged 15 to 64 living in the European Union is of 0.6% and increases to 1.1% when considering individuals aged 15 to 34 [9]. According to the Flash Eurobarometer survey, the lifetime prevalence of NPS use among young people (15–24 y.o.) is of 8% in 2014, compared to 5% in 2011; focusing on the Italian population, the prevalence is of 6% [11]. The rapid expansion and distribution of NPS is challenging, in the medical context, due to unclear toxicological effects and the serious health risks frequently associated with their consumption. Given that NPS are commercialized as “legal” substitutes for illegal recreational drugs, there is a perception that these are less dangerous, and individuals may consume them thinking they are not merely legal, but also medically safe [12,13]. Nevertheless, these new drugs can produce strong psychoactive effects, acute intoxication, and serious medical complications, such as seizures, strokes, cardiovascular toxicity, renal failure, hyperthermia, respiratory depression, loss of consciousness, delirium, psychosis, and many others [1,3,14,15,16]. Furthermore, NPS have the potential to produce strong withdrawal syndromes, tolerance, and addiction [17,18,19], and they are related to an increased risk of developing severe infectious diseases, such as HIV and HCV [20,21]. Over the years, there has been an increase in emergency room visits related to NPS acute intoxication, as well as a corresponding increase in fatalities, due to overdose or accidents related to the NPS intoxication [9,22].

While some international studies have investigated the prevalence, characteristics, and factors associated with use of NPS, both in the general population and specific subpopulations, these topics remain unexplored in the Italian research framework. Existing data have mostly been collected from surveys sponsored by the European Commission. According to the Italian version of the European School Survey Project on Alcohol and other Drugs (ESPAD), 4.1% of young individuals (15–19 y.o.) have tried NPS at least once in their life [23]. Only a few Italian studies have examined the use of NPS in the general population or specific populations of young individuals and psychiatric patients [24,25]. To the best of our knowledge, there is no available data on the use of NPS in users of drug addiction services in Italy. Examining the use of NPS in a clinical sample of individuals with substance-related disorders could generate evidence to understand the prevalence of NPS use, as well as its clinical correlates and patients’ perceptions on NPS toxicity, uncovering an underestimated potentially clinical phenomenon.

Therefore, the aim of the present study is to determine knowledge and frequency of use of NPS and develop a comprehensive account of the characteristics and clinical correlates associated with the use of the NPS in an Italian sample of drug addiction services patients.

## 2. Materials and Methods

### 2.1. Participants

Subjects were recruited through three main local addiction services “Servizio Dipendenze” (Ser.D) of Padova, Belluno, and Feltre, Italy. Other Ser.D involved were: Chioggia, Dolo, Mestre, Monfalcone, Montebelluna, Palmanova, Trieste, Udine, Vicenza, and Verona. Patients gave their informed consent to participate to the survey. The sample was composed of 185 active users of the services with a diagnosis of substance use disorder (SUD), as per ICD-9. In order to be included in the study, participants must be aged 18 to 45 and followed by the Ser.D for problems related to the use of illegal substances. Subjects were taking methadone, in the context of the agonist treatment program.

### 2.2. Procedure

During a period of five months, from February 2019 to July 2019, subjects were recruited and asked to anonymously complete an ad-hoc questionnaire, available in its English version in the Supplementary Material File S1. Each participant was fully informed about the aims of the study and, after confirming they met inclusion criteria, brought to a private room to complete the questionnaire anonymously. The items of the questionnaire were read to each participant and responses were recorded on an answer sheet by the researcher. Study procedures were previously approved by the corresponding local health authority (i.e., AULSS 6 Euganea and AULSS 1 Dolomiti).

### 2.3. Questionnaire

We used a questionnaire designed ad-hoc to fulfil the objectives of the present study, which was composed of 63 items, divided into three sections: the first 20 items collect demographic data, items 21 to 24 assess the use of traditional drugs and the associated perceived risk, and the remaining items explore knowledge and use of NPS. In particular, item 25 asks whether the subject has ever used one of the listed NPS, has never used them but knows of them, or has never used them and does not know of them. If the subject reports use of NPS, he/she will be asked to specify the type (i.e., synthetic cannabinoids, synthetic cathinones, depressant NPS (such as opioids and benzodiazepines, hallucinogenic, and dissociative NPS), or NPS of natural origin, according to classification in Table 1), whether the NPS was used over the last 30 days, and the age of first consumption.

The subject was also asked to answer questions related to the specific substance used (i.e., between item 29 to 58) and specify the main route of drug administration, frequency of use, place of consumption, mode of acquisition, and primary motivation for use. Information about the concomitant use of alcohol and NPS, as well as the perceived risk associated with NPS use, were also assessed. If the subject responded that they know of NPS but have never used them, he/she would answer items 59 to 63 about how the NPS was known, legal and health risk perception, willingness to take the NPS, and potential motivations for doing so.

### 2.4. Statistical Analysis

Questionnaire responses were transferred to a spreadsheet and analyzed using “Jamovi” statistical software (Jamovi, Sydney, Australia). Descriptive analyses were conducted, in order to characterize the study sample, as well as comparative and correlational analyses to identify factors potentially associated with NPS use. Results with a *p* < 0.05 were considered statistically significant.

## 3. Results

We collected data from 185 service users of which *n* = 123 (66.5%) were recruited at the Ser.D of Padova, *n* = 21 (11.4%) at the Ser.D of Belluno, *n* = 25 (13.5%) at the Ser.D of Feltre, and *n* = 16 (8.6%) at other Italian Ser.D (i.e., Chioggia, Dolo, Mestre, Monfalcone, Montebelluna, Palmanova, Trieste, Udine, Vicenza, and Verona).

The mean age of participants was 31.2 ± 7.78 years. Study participants were 83.8% (*n* = 155) male and 16.2% (*n* = 30) female. A total of 45.9% of subjects were aged 25 to 35, with 30.8% aged 36 to 45. Italian nationals comprised the majority of the study population at 79.5% (*n* = 147), with the remaining 20.5% (*n* = 38) self-identifying as foreign nationals. The mean age at which the subjects had their first contact with the addiction services was 24.3 ± 6.62, with a minimum age of 12 and maximum age of first contact of 44. Table 2 presents the demographic features of the sample organized by local addiction service.

Regarding NPS, 33.5% (*n* = 62) of survey subjects reported having used at least one NPS in his/her life. Of the remaining 66.5% (*n* = 123), 31.4% (*n* = 58) were aware of NPS.

As expected in a SUD clinical sample, methadone was reported as the most commonly used substance in the study sample (94.6%; *n* = 175), followed by heroin (91.9%; *n* = 170) and THC (90.8%; *n* = 168). NPS of natural origin were self-reported as the most commonly used NPS in our sample (21.6%; *n* = 40), followed closely by synthetic cannabinoids (17.3%; *n* = 32) and depressant NPS (14.1%; *n* = 26). The mean age of first use of traditional substances was 19.2 ± 4.74 and NPS was 19.1 ± 4.0. Table 3 shows the frequency of use and mean age of first use of each substance.

We conducted correlational analyses to identify factors potentially associated with NPS use. First, a polydrug use analysis on the concomitant consumption of NPS and traditional drugs was conducted. The use of most traditional drugs was correlated with the use of NPS; in particular, cocaine (OR = 14.0; *p* = 0.011), THC (OR = 11.6; *p* = 0.034), LSD (OR = 15.6; *p* < 0.001), MDMA (OR = 9.52; *p* < 0.001), amphetamine (OR = 12.1; *p* < 0.001), methamphetamine (OR = 6.78; *p* < 0.001), or drugs without a prescription (OR = 5.11; *p* < 0.001) were all related to use of NPS.

Clinical and demographic features, related to the use of NPS, were also taken into consideration. In our sample, the use of NPS was correlated with youth (i.e., 18–24 y.o.) (OR = 4.81; *p* < 0.001), student status (OR = 4.99; *p* < 0.001), living in a residential setting (OR = 5.66; *p* = 0.002), history of attempted suicide (OR = 11.67; *p* < 0.001), diagnosis of a mental disorder after substance use (OR = 2.49; *p* < 0.001), and being on a house detention regime (OR = 2.30; *p* < 0.001). Factors associated with NPS are reported in Table 4.

Finally, comparative analyses were computed to examine differences between risk perception and polydrug abuse of both traditional and NPS. In general, study participants believed that NPS are less dangerous than traditional drugs (*p* < 0.001). In our SUD population, those who had consumed NPS used a wider variety of drugs, compared to participants who had used only traditional drugs of abuse (*p* < 0.001), with a mean of 7.94 substances used for those that consume NPS and a mean of 5.29 substances used for those who do not consume NPS. Table 5 presents the differences in risk perception and polydrug use between NPS and traditional substances.

Finally, we investigated the motivations to consume drugs, only for what concerns NPS and not for traditional drugs, with a multiple-choice question. The principal motivation for using the NPS was to get high (41.9%). Other recurrently cited motivations to use the NPS were for novelty/curiosity (17.8%), to have fun (13.2%), to relax (11.6%), and to forget problems (7.8%). Other minor motivations were the perception of less legal risks (compared to the “traditional substances”) (3.1%), the perception of less health dangerousness (compared to the “traditional substances”) (2.3%), and to get increased effects of other substances (2.3%)”.

## 4. Discussion

The present study examined the use of NPS and associated factors in a specific population of addiction service users in the north-eastern area of Italy. While the majority of the sample population was being followed for SUD related to traditional drugs of abuse (i.e., methadone, heroin, and THC), more than a third of participants reported use of NPS and another third reported awareness of the latter. The lifetime prevalence of NPS use among drug users in this sample is comparable to results of other studies conducted in Europe to date [47,48].

We were able to identify factors correlated with the use of NPS in a clinical sample. First, being a polydrug user was a risk factor for the use of NPS. Subjects who used NPS reported consuming a higher number of drugs overall, compared to individuals who used only traditional drugs. These results are in line with previous findings, suggesting that NPS use is associated with polysubstance abuse [49,50]. In our study, population youth and, relatedly, student status were strongly associated with NPS use. This aligns with prior research showing that NPS use is more frequent among younger individuals both in the general population [11,23] and specific clinical populations [49]. Other variables shown to be associated with NPS use in the present study were previous suicide attempts and being diagnosed with a psychiatric disorder. According to the literature, those that consume synthetic cannabinoids and other high potency NPS have higher rates of psychiatric distress [49], mainly in the area of schizophrenia spectrum and bipolar disorders [51,52]. Moreover, a relatively new Italian study showed how young psychiatric patients tend to consume higher rates of NPS, compared to their healthy counterparts [24], posing a risk for medical and psychopathological sequalae [53,54]. While substance use is common among individuals with psychiatric diagnoses, it is notable that the consumption of NPS is also increasing in this at-risk population of adolescents and young adults.

We found that individuals who reside in residential settings and have legal issues, such as being under house arrest, were also more likely to have used NPS. According to a European report, an increasing number of countries report the use of NPS in prison settings, including Italy [55]. Individuals with legal issues may be more likely to use NPS, given they are often undetectable by the drug screening, to which individuals in the legal setting are often regularly subjected, as a condition of their release into the community.

NPS are mostly consumed by more vulnerable service users, such as younger individuals, polydrug users, and patients with other psychiatric conditions, potentially contributing to a worsening of their overall prognosis. In addition, use of NPS is made increasingly likely, given they are perceived as being less dangerous, compared to traditional drugs of abuse. Indeed, studies have reported that one of the main reasons patients cite for consuming NPS is their safer profile [49,56]. With newly synthetized compounds being continuously introduced into the drug market, it is immensely challenging for health professionals to keep track of the potential adverse effects these compounds may produce, both in intoxication states and withdrawal. Clinicians should be aware of the existence of the categories of NPS drugs and expected toxidromes of each, as well as the fact that the field of NPS is expanding rapidly. Given the elevated risk of NPS use in the SUD treatment program population, specialists in SUD treatment should make particular efforts to stay up to date on newly identified drugs and their toxidromes as evidence becomes available. Whenever possible, health professionals should inform addiction service users of the potential risks of addiction and toxicity that may be associated with NPS use.

There are a number of limitations in the current study. Data were collected through a self-reported questionnaire and may not accurately reflect participant’s experiences with NPS. While there would be no clear motivation to do so, participants with legal issues may not feel comfortable disclosing NPS use and may have under-reported. In polydrug users, there may be significant issues with recall of the timing or frequency of prior use. Furthermore, the ad-hoc questionnaire used to fulfil the objectives of the study is not a validated instrument of measurement, and the resultant data may not be replicable in future studies. Additionally, we did not report specific ICD/DSM diagnoses, given the self-report nature of the study, in terms of specific substance used. All patients, however, met the ICD-9 criteria for a substance use disorder. In addition, methadone was prescribed as a replacement treatment for opioids addiction. Finally, the present study utilized a cross-sectional design and surveyed a non-representative sample of addiction service users, within which a majority of respondents were male, limiting inference on the female population. Our results are, therefore, not able to be used to make inferences about the general population.

Future research should consider using validated and objective tools to assess the prevalence of NPS use (i.e., hair and urine samples) and expand the investigation adopting a longitudinal study design, recruiting a more representative sample of individuals with SUD, including users of other Italian services.

## 5. Conclusions

NPS are a serious public concern, involving both the general population and at-risk subpopulations. The present study is the first investigating the use of NPS and its correlates in an Italian sample of individuals with substance-related disorders. Evaluating patterns of NPS use and identifying high-risk individuals is an important first step in the effort to manage this burgeoning public health issue. Based on the results of the present study, we conclude that the use of NPS is diffuse amongst addiction service users. We found that two thirds of the participants knew about NPS and one third have used them at some point in their life. We also identified the factors associated with an increased likelihood of consumption of NPS, such as polydrug use, younger age, studentship, suicidal attempt, other psychiatric diagnosis, residential care, and legal issues. In this study, individuals that are more likely to use NPS also happen to be those that are already more vulnerable and suffer poor mental and physical health outcomes at baseline. The use of NPS likely worsens their treatment prognosis and lengthens time to recovery, but further research in this area is needed. NPS are generally perceived as safer than more established illegal substances, but these new compounds have not been extensively researched. Given the wide and rapidly increasing variety of NPS drugs available for consumption, each with relatively unknown adverse effects, habit-forming potential, withdrawal symptoms, and toxidromes in overdose, the use and misuse of NPS presents a huge challenge to the healthcare system and may silently contribute to untold fatalities in the emergency setting. Health professionals should be better informed on how to identify NPS use and supervise drug users that may be at-risk of consuming NPS.

## Figures and Tables

**Table 1:** Novel psychoactive substances (NPS) and their effects.

Class NPS	Effects	Psychiatric Risks	Medical Risks	Example NPS
Synthetic cannabinoids	Intoxicant, stimulant; cannabimetics effect;	Paranoia; agitation; confusion; hallucinations, psychosis; addiction; cognitive impairment [26,27];	Tachycardia; hypertension; myocardial infarction; renal failure; pulmonary damage [26,28];	Spice drugs; “K2”; HU-210; JWH-018;
Synthetic cathinones	Stimulant;	Insomnia; euphoria; irritability, visual hallucinations; anxiety; hypervigilance; psychotic symptoms; delirium; impulsive behaviour; suicide; agitation; dysphoria; amnesia; anhedonia [16,29,30,31,32];	Involuntary muscle clenching; hyperthermia; tachycardia; nausea and vomiting; cardiovascular toxicity; renal and respiratory failure; rash; stroke; death [16,29,30,31];	Mephedrone; MDPV; PMA/PMMA; pFBT
Depressant NPS (opioids; benzodiazepines)	Sedative; anxiolytic; hypnotic;	Confusional states, seizures after withdrawal; addiction; impaired cognition; persistent hallucinations; amnesia [33,34,35];	Bradycardia; sedation; vomiting; seizures after withdrawal; respiratory failure; death [35,36];	Fentanyl analogues; AH- 7921; U47700; MT 45; diclazopam; flubromazepam;
Hallucinogenic and dissociatives NPS	Hallucinogenic-psychedelic effect; dissociation; sensory deprivation	Hallucinations; amnesia; delirium; increased impulsivity; anti-social like behaviours; improved mood; panic attacks; paranoid thoughts; fatal accidents; agitation; confusion; aphasia; aggressive/psychotic states; memory/mood problems; paranoia; euphoria [37,38,39,40,41,42];	Hypertension; sweating; tachycardia; involuntary eye movement; poor coordination; peripheral vasoconstrictor; necrosis; kidney failure; respiratory failure [37,40,41,42,43];	5-MeO-DALT; NBOMe-series; 2C-series; Methoxetamine;
Natural NPS	Hallucinogenic-psychedelic effect; dissociation; sensory deprivation; stimulant	Persistent hallucination; insomnia; agitation; irritability; confusion; dissociation; sensory deprivation; paranoia; psychosis; depression; aggressive/psychotic states; memory/mood problems; paranoia [44,45,46].	Tachycardia; visual failure; stroke; dehydration; sweating; vasospasms; esophagitis; gastritis; and hepatitis [44,45,46].	Khat; Kratom; Ayahuasca (DMT); Mushrooms; *Salvia Divinorum*

**Table 2:** Description of the sample including demographic features.

*N* = 185			
Addiction Services	Demographic Features	*N*/Mean	%/SD
Ser.D of Padua *n* = 123			
Gender	M	100	81.3%
	F	23	18.7%
Age		30.8	8.03
Nationality	Italian	86	69.9%
	Other	37	30.1%
Ser.D 1st time age		24.6	6.74
Ser.D of Belluno *n* = 21			
Gender	M	21	100%
Age		33.3	7.79
Nationality	Italian	21	100%
Ser.D 1st time age		23.2	4.92
Ser.D of Feltre *n* = 25			
Gender	M	20	80%
	F	5	20%
Age		31.4	7.54
Nationality	Italian	25	100%
Ser.D 1st time age		24.6	8.18
Other italian Ser.D * *n* = 16			
Gender	M	14	87.5%
	F	2	12.5%
Age		31.3	6.22
Nationality	Italian	15	93.8%
	Other	1	6.3%
Ser.D 1st time age		23.2	4.97

* Chioggia, Dolo, Mestre, Monfalcone, Montebelluna, Palmanova, Trieste, Udine, Vicenza, and Verona.

**Table 3:** Distribution and age of subjects using different substances.

	% Total	*N*	Age Mean (SD)
Traditional substances			
Methadone	94.6	175	23.7 (6.6)
Heroin	91.9	170	19.5 (5.2)
THC	90.8	168	16.7 (4)
Cocaine	87	161	19.2 (5)
LSD	63.8	118	18.8 (4)
MDMA	54.1	100	18.4 (3.9)
Amphetamine	53	98	18.8 (4.8)
Drugs without prescription *	47	87	19.1 (4.8)
Methamphetamine	35.7	66	18.3 (4.4)
NPS			
Natural NPS	21.6	40	19.7 (19.5)
CBN NPS **	17.3	32	19.9 (20)
Depressant NPS	14.1	26	20.3 (19.5)
Hallucinogenic/dissociatives NPS	9.7	18	19.2 (18)

* Any medicine taken by the participants, for which they had no medical prescription, such as benzodiazepines, morphine, buprenorphine, or methadone. ** Synthetic cannabinoids.

**Table 4:** Factors associated with use of novel psychoactive substances (NPS).

**NPS Polyabuse Analysis (*n* = 62)**			**OR**	**Confidence Intervals 95%**
NPS–Cocaine	%NPS and cocaine use (*n* = 61) %NPS and no cocaine use (*n* = 1)	37.9% 4.2%	14.0	1.8–106.5
NPS–THC	%NPS and THC use (*n* = 61) %NPS and no THC use (*n* = 1)	36.3% 5.9%	11.6	4.35–30.9
NPS–LSD	%NPS and LSD use (*n* = 57) %NPS and no LSD use (*n* = 5)	48.3% 7.5%	15.6	1.2–70.5
NPS–MDMA	%NPS and MDMA use (*n* = 53) %NPS and no MDMA use (*n* = 9)	53.0% 10.6%	9.52	4.30–21.1
NPS–Amphetamine	%NPS and amphetamine use (*n* = 54) %NPS and no amphetamine use (*n* = 8)	55.1% 9.2%	12.1	5.29–27.8
NPS–Metamphetamine	%NPS and metamphetamine use (*n* = 40) %NPS and no metamphetamine use (*n* = 22)	60.6% 18.5%	6.78	3.45–13.3
NPS–Drugs without a prescription	%NPS and drugs without a pres.use (*n* = 45) %NPS and no drugs without a pres.use (*n* = 17)	51.7% 17.3%	5.11	2.61–9.99
**Clinical and Demographic Features Analysis (*n* = 62)**			**OR**	**Confidence Intervals 95%**
NPS use–Addiction services reference = Ser.D Padua (*n* = 30); OR 0.32 (0.21–0.49) *p* < 0.001 vs others	%NPS and Ser.D Belluno (*n* = 8) %NPS and Other Addiction Services (*n* = 24)	38.1% 58.5%	1.91 4.38	0.72–5.04 2.08–9.22
NPS use–Ages range reference = range 36 + (*n* = 11); OR 0.29 (0.12–0.46) *p* < 0.001 vs others	%NPS and range 18–24 (*n* = 23) %NPS and range 25–35 (*n* = 28)	53.5% 32.9%	4.81 2.54	1.98–11.71 0.93–4.56
NPS use–Occupation reference = employed (*n* = 17); OR 0.32 (0.18–0.54) *p* < 0.001 vs others	%NPS and unemployed (*n* = 34) %NPS and students (*n* = 11)	35.4% 61.1%	1.74 4.99	0.88–3.46 1.67–15.89
NPS use–Setting reference = outpatient (*n* = 50); OR 0.42 (0.30–0.59) *p* < 0.001 vs others	%NPS in residential setting (*n* = 12)	70.6%	5.66	1.90–16.92
NPS use–Suicide Attempt reference = no suicide attempt (*n* = 48); OR 0.40 (0.29–0.56) *p* < 0.001 vs others	%NPS and suicide attempt (*n* = 14)	82.4%	11.67	3.21–42.43
NPS use–Psychiatric diagnosis after use substance reference = no p.s. after use (*n* = 47); OR 0.43 (0.31–0.61) *p* < 0.001 vs others	%NPS and psych. diagnosis after use (*n* = 15)	51.7%	2.49	1.11–5.56
NPS use–House detention reference = no house detention (*n* = 47); OR 0.44 (0.31–0.61) *p* < 0.001 vs others	%NPS and house detention (*n* = 15)	50.0%	2.30	1.04–5.08

**Table 5:** Differences in risk perception and polydrug use between NPS and traditional substances.

	Traditional Substance Use Mean	NPS Use Mean	T	*p*-Value
Risk Perception (interval 1–5)	4.18	3.40	6.061	*p* < 0.001
Polydrug use (interval 2–9)	5.29	7.94	8.985	*p* < 0.001

## Data Availability

The data presented in this study are available on request from the corresponding author. The data are not publicly available due to privacy.

## Supplementary Materials

The following supporting information can be downloaded at: https://www.mdpi.com/article/10.3390/ijerph19020915/s1, File S1: supplementary material.
