# Supplementary material for: Knowledge and Use of Novel Psychoactive Substances in an Italian Sample with Substance Use Disorders
Source: Int J Environ Res Public Health. 2022 Jan 14;19(2):915. doi: 10.3390/ijerph19020915 (PMC8776073; doi:10.3390/ijerph19020915)
Supplement: Supplementary file 1 [file ijerph-19-00915-s001.zip › ijerph-1488096-supplementary.pdf]

SUPPLEMENTARY MATERIAL

Questionnaire NPS

N ___

Date __/__/____ Place _____________________

1. Age _________

2. Sex M F

3. Nationality ______________________________

4. If you are a foreign national, at what age did you arrive in Italy? ____

5. Place of residence (indicate the province) ______________________________

6. Which Ser. D do you belong to? ______________________________

7. Highest educational qualification obtained:
   - None
   - Elementary school degree
   - Middle school degree
   - High school degree
   - Bachelor's degree or Master's degree

8. What is your occupation:
   - Employed
   - Unemployed
   - Looking for occupation
   - Housewife
   - Student

9. Marital status:
   - Unmarried
   - Married
   - Separated
   - Divorced
   - Widowed

10. Who do you live with?
    - Alone
    - With parents
    - With a partner
    - With friends/housemates

11. What is the age of your first contact with a service for the drug addiction treatment? (e.g., Ser.D, residential program, hospitalization) ______

12. Current treatment:
- Outpatient rehab program
- Residential rehab program
- Other

13. Are you currently on a house detention regime, with a suspended sentence or with an alternative sanction?
- Yes
- No

14. Where you diagnosed with a mental disorder **before** starting to use drugs?
- Yes (specify ______________________________)
- No

15. Where you diagnosed with a mental disorder **during or after** your addiction?
- Yes (specify ______________________________)
- No

16. Where you prescribed any medication to treat your mental disorder(s)?
- Yes (specify______________________________)
- No

17. Did you attempt suicide?
- Yes
- No

18. *Only if you answered Yes to the previous question*, where you hospitalized at the psychiatric services after the suicide attempt?
- Yes (specifiy the year ______)
- No

19. Do you have any severe physical disease that interferes with your life?
- Yes (specifiy ______________________________)
- No

20. *Only if you answered Yes to the previous question*, do you take any medication to treat your physical disease(s)?
- Yes (specifiy ______________________________)
- No

21. Which of the following psychoactive substances did you use in your life? Draw an X in the specified column if you tried the substance AT LEAST ONCE in your life (you can indicate multiple substances), then specify if you consumed the specific substance during the last 30 days and the age of first use.

	At least once	Last 30 days	Age of first use
Heroine	_____	_____	_____
Methadone	_____	_____	_____
Cocaine, crack	_____	_____	_____

Cannabinoids (marijuana, hashish)	____	____	____
MDMA (ecstasy)	____	____	____
Amphetamine	____	____	____
Metamphetamine ("shaboo")	____	____	____
LSD or other psychedelics (GHB, GBL), dissociative substances such as Ketamine and PCP	____	____	____
Drugs without a medical prescription (e.g., Methadone, benzodiazepines, morphine)	____	____	____

22. Have you ever used any substance while drinking alcohol?

- Yes
- No

23. *Only if you answered Yes to the previous question*, what is the frequency with which you simultaneously consume/consumed drugs and alcohol?

- Everyday
- More than once in a week
- 2-3 times a week
- I have only tried one or two times in my life

24. Do you think that serious consequences for your physical or mental health could result from the consumption of the aforementioned drugs?

- Not at all
- A little
- Moderately
- A lot
- Extremely

25. Have you ever tried the so called "smart drugs"? Draw an X in the specified column if you tried the substance AT LEAST ONCE in your life (you can indicate multiple substances), then specify if you consumed the specific substance during the last 30 days and the age of first use.

	At least once	Last 30 days	Age of first use
**Synthetic cannabinoids** (known as Spice, Black mamba, Pandora's box, Arancia meccanica, K2, X, Tai high, Hawaiian haze, Mary joy, Devil's weed)	__	___	____
**Synthetic cathinones** derived from Khat, such as Mephedrone, Flakka and methylone (sold as "bath salts" or "plant fertilizers"), MDPV ("Super coke") and APVP	__	___	____
**Synthetic opioids** derived from Fentanyl, AH-7921, MT-45, and new benzodiazepines such as Diclazepam and Flubromazepam	__	___	____
**Synthetic hallucinogens** such as Phenylethylamine, 5-Meo-DALT, 2C-series, NBOMe-series, and dissociative substances such as Methoxetamine ("Mexxy")	__	___	____

**Substances of natural origin** such as Kratom, Ayahuasca, Khat and *Salvia divinorum*	__	___	____
I have never used these substances, but I know them	__	___	____
I have never used these substances, and I DON'T know them	__	___	____

26. Have you ever used any "smart drug" while drinking alcohol?
- Yes
- No

27. *Only if you answered Yes to the previous question*, what is the frequency with which you simultaneously consume/consumed smart drugs and alcohol?
- Everyday
- More than once a week
- 2-3 times a week
- I have only tried one or two times in my life

28. Do you think that serious consequences for your physical or mental health could result from the consumption of the aforementioned "smart drugs" ?
- Not at all
- A little
- Moderately
- A lot
- Extremely

ANSWER TO THE FOLLOWING QUESTIONS ONLY IF YOU TREID THE ***SYNTHETIC CANNABINOIDS*** *(known as Spice, Black mamba, Pandora's box, Arancia meccanica, K2, X, Tai high, Hawaiian haze, Mary joy, Devil's weed) AT LEAST ONCE IN YOUR LIFE.*

29. Can you specify your most frequent mode of administration of the drug?
    - Oral (e.g., tablets or pills)
    - Nasal (e.g., sniffing)
    - Smoked
    - Injected
    - Rectal

30. Can you specify the frequency of use?
    - Everyday
    - More than once a week
    - 2-3 times a week or during weekends
    - Only 1 or 2 times in my life

31. Where did you mostly consumed the drugs?
    - Outside (at the park, in the street, at the beach, at the train station, …)
    - At the workplace or at school
    - At your home or a friend's home
    - At the disco or clubs
    - During a party or a rave party

32. Who provided you the drug most often?
    - Offered by friends
    - Offered by a stranger
    - Bought

33. *Only if you bought the drug*, where did you principally buy it?
    - Outside (at the park, in the street, at the beach, at the train station, …)
    - At the workplace or at school
    - At a friend's home
    - At the disco or clubs
    - During a party or a rave party
    - From a drug dealer
    - Smart shop
    - Through websites or social networks (online smart shop, online pharmacies, commercial ads, marketplace...)

34. What is the principal motivation that encouraged you to consume these substances?
    - To get high
    - To have fun with friends
    - To relax
    - To forget the problems
    - To perform better at school or at work
    - To perform better at sports or during sex
    - Curiosity and novelty
    - For the increased effects
    - Perception of less legal risks compared to the "traditional substances"
    - Perception of less health dangerousness compared to the "traditional substances"

ANSWER TO THE FOLLOWING QUESTIONS ONLY IF YOU TREID THE ***SYNTHETIC CATHINONES*** *(derived from Khat, such as Mephedrone, Flakka and methylone (sold as "bath salts" or "plant fertilizers"), MDPV ("Super coke") and APVP) AT LEAST ONCE IN YOUR LIFE.*

35. Can you specify your most frequent mode of administration of the drug?
    - Oral (e.g., tablets or pills)
    - Nasal (e.g., sniffing)
    - Smoked
    - Injected
    - Rectal

36. Can you specify the frequency of use?
    - Everyday
    - More than once a week
    - 2-3 times a week or during weekends
    - Only 1 or 2 times in my life

37. Where did you mostly consumed the drugs?
    - Outside (at the park, in the street, at the beach, at the train station, …)
    - At the workplace or at school
    - At your home or a friend's home
    - At the disco or clubs
    - During a party or a rave party

38. Who provided you the drug most often?
    - Offered by friends
    - Offered by a stranger
    - Bought

39. *Only if you bought the drug*, where did you principally buy it?
    - Outside (at the park, in the street, at the beach, at the train station, …)
    - At the workplace or at school
    - At a friend's home
    - At the disco or clubs
    - During a party or a rave party
    - From a drug dealer
    - Smart shop
    - Through websites or social networks (online smart shop, online pharmacies, commercial ads, marketplace...)

40. What is the principal motivation that encouraged you to consume these substances?
    - To get high
    - To have fun with friends
    - To relax
    - To forget the problems
    - To perform better at school or at work
    - To perform better at sports or during sex
    - Curiosity and novelty
    - For the increased effects
    - Perception of less legal risks compared to the "traditional substances"
    - Perception of less health dangerousness compared to the "traditional substances"

ANSWER TO THE FOLLOWING QUESTIONS ONLY IF YOU TREID THE ***SYNTHETIC OPIOIDS*** *(derived from Fentanyl, AH-7921, MT-45, and new benzodiazepines such as Diclazepam and Flubromazepam) AT LEAST ONCE IN YOUR LIFE.*

41. Can you specify your most frequent mode of administration of the drug?
    - Oral (e.g., tablets or pills)
    - Nasal (e.g., sniffing)
    - Smoked
    - Injected
    - Rectal

42. Can you specify the frequency of use?
    - Everyday
    - More than once a week
    - 2-3 times a week or during weekends
    - Only 1 or 2 times in my life

43. Where did you mostly consumed the drugs?
    - Outside (at the park, in the street, at the beach, at the train station, …)
    - At the workplace or at school
    - At your home or a friend's home
    - At the disco or clubs
    - During a party or a rave party

44. Who provided you the drug most often?
    - Offered by friends
    - Offered by a stranger
    - Bought

45. *Only if you bought the drug*, where did you principally buy it?
    - Outside (at the park, in the street, at the beach, at the train station, …)
    - At the workplace or at school
    - At a friend's home
    - At the disco or clubs
    - During a party or a rave party
    - From a drug dealer
    - Smart shop
    - Through websites or social networks (online smart shop, online pharmacies, commercial ads, marketplace...)

46. What is the principal motivation that encouraged you to consume these substances?
    - To get high
    - To have fun with friends
    - To relax
    - To forget the problems
    - To perform better at school or at work
    - To perform better at sports or during sex
    - Curiosity and novelty
    - For the increased effects
    - Perception of less legal risks compared to the "traditional substances"
    - Perception of less health dangerousness compared to the "traditional substances"

ANSWER TO THE FOLLOWING QUESTIONS ONLY IF YOU TREID THE ***SYNTHETIC HALLUCINOGENS*** *(such as Phenylethylamine, 5-Meo-DALT, 2C-series, NBOMe-series, and dissociative substances such as Methoxetamine ("Mexxy")) AT LEAST ONCE IN YOUR LIFE.*

47. Can you specify your most frequent mode of administration of the drug?
- Oral (e.g., tablets or pills)
- Nasal (e.g., sniffing)
- Smoked
- Injected
- Rectal

48. Can you specify the frequency of use?
- Everyday
- More than once a week
- 2-3 times a week or during weekends
- Only 1 or 2 times in my life

49. Where did you mostly consumed the drugs?
- Outside (at the park, in the street, at the beach, at the train station, …)
- At the workplace or at school
- At your home or a friend's home
- At the disco or clubs
- During a party or a rave party

50. Who provided you the drug most often?
- Offered by friends
- Offered by a stranger
- Bought

51. *Only if you bought the drug*, where did you principally buy it?
- Outside (at the park, in the street, at the beach, at the train station, …)
- At the workplace or at school
- At a friend's home
- At the disco or clubs
- During a party or a rave party
- From a drug dealer
- Smart shop
- Through websites or social networks (online smart shop, online pharmacies, commercial ads, marketplace...)

52. What is the principal motivation that encouraged you to consume these substances?
- To get high
- To have fun with friends
- To relax
- To forget the problems
- To perform better at school or at work
- To perform better at sports or during sex
- Curiosity and novelty
- For the increased effects
- Perception of less legal risks compared to the "traditional substances"
- Perception of less health dangerousness compared to the "traditional substances"

ANSWER TO THE FOLLOWING QUESTIONS ONLY IF YOU TREID THE ***SUBSTANCES OF NATURAL ORIGIN*** *(such as Kratom, Ayahuasca, Khat and Salvia divinorum) AT LEAST ONCE IN YOUR LIFE.*

53. Can you specify your most frequent mode of administration of the drug?
    - Oral (e.g., tablets or pills)
    - Nasal (e.g., sniffing)
    - Smoked
    - Injected
    - Rectal

54. Can you specify the frequency of use?
    - Everyday
    - More than once a week
    - 2-3 times a week or during weekends
    - Only 1 or 2 times in my life

55. Where did you mostly consumed the drugs?
    - Outside (at the park, in the street, at the beach, at the train station, …)
    - At the workplace or at school
    - At your home or a friend's home
    - At the disco or clubs
    - During a party or a rave party

56. Who provided you the drug most often?
    - Offered by friends
    - Offered by a stranger
    - Bought

57. *Only if you bought the drug*, where did you principally buy it?
    - Outside (at the park, in the street, at the beach, at the train station, …)
    - At the workplace or at school
    - At a friend's home
    - At the disco or clubs
    - During a party or a rave party
    - From a drug dealer
    - Smart shop
    - Through websites or social networks (online smart shop, online pharmacies, commercial ads, marketplace...)

58. What is the principal motivation that encouraged you to consume these substances?
    - To get high
    - To have fun with friends
    - To relax
    - To forget the problems
    - To perform better at school or at work
    - To perform better at sports or during sex
    - Curiosity and novelty
    - For the increased effects
    - Perception of less legal risks compared to the "traditional substances"
    - Perception of less health dangerousness compared to the "traditional substances"

ANSWER TO THE FOLLOWING QUESTIONS ONLY IF YOU HAVE ***NEVER*** *USED "SMART DRUGS", BUT YOU KNOW THEM.*

59. How did you get to know these substances?
- Friends or acquaintances
- Traditional mass media (tv, journals, newspapers, radio)
- Social network or smartphone apps
- During a party or a rave party
- Websites or online communities

60. Do you think that the "smart drugs" entail less legal risks compared to the "traditional substances"?
- Yes
- No

61. Do you think that the "smart drugs" are less dangerous for your physical and mental health compared to the "traditional substances"?
- Yes
- No

62. Are you going to try the "smart drugs" at least once?
- Yes
- No

63. *Only if you answered Yes to the previous question*, why would you like to try them?
- To get high
- To have fun with friends
- To relax
- To forget the problems
- To perform better at school or at work
- To perform better at sports or during sex
- Curiosity and novelty
- For the increased effects
- Perception of less legal risks compared to the "traditional substances"
- Perception of less health dangerousness compared to the "traditional substances"
